# The relationship between non-HDL-C/HDL-C ratio and bone mineral density: an NHANES study

**DOI:** 10.3389/fnut.2024.1486370

**Published:** 2025-01-07

**Authors:** Shuo Qi, Biao Peng, Zhanwang Xu, Daodi Qiu, Guoqing Tan

**Affiliations:** ^1^The First Clinical Medical College of Shandong University of Traditional Chinese Medicine, Jinan, Shandong, China; ^2^Department of Pulmonary and Critical Care Medicine, The Affiliated Changsha Central Hospital, Hengyang Medical School, University of South China, Changsha, Hunan, China; ^3^Department of Spinal and Spinal Cord, Affiliated Hospital of Shandong University of Traditional Chinese Medicine, Jinan, Shandong, China

**Keywords:** the non-high-density lipoprotein cholesterol to high-density lipoprotein cholesterol ratio, BMD, NHANES, a cross-sectional study, lipid

## Abstract

**Background:**

The non-high-density lipoprotein cholesterol to high-density lipoprotein cholesterol ratio (NHHR) is a newly developed lipid parameter. However, the current research has only explored the relationship with lumbar spine bone mineral density, lacking studies on bone mineral density at other sites, total body bone mineral density, and an analysis of risk factors. This study aims to determine the potential association between NHHR and lumbar BMD, increase awareness of the impact of lipid levels on bone health.

**Methods:**

By utilizing data from the National Health and Nutrition Examination Survey (NHANES) from 2011 to 2018, we conducted univariate and generalized linear models (GLMs) analysis, stratified analysis, threshold effect analysis, smooth curve fitting and stratified analysis to investigate the association between NHHR and BMD. NHHR levels were categorized into tertiles (low, medium, and high) based on their distribution among the study population.

**Results:**

The study included 8,671participants, studies have shown, the ratio of non-high-density lipoprotein to high-density lipoprotein (NHHR) exhibits a stratified correlation with bone mineral density (BMD). In the BMI subgroup, NHHR is significantly negatively correlated with BMD at multiple sites in the low-to-middle BMI group (BMI <25 kg/m^2^), while no significant correlation is found in the high BMI group (BMI ≥30 kg/m^2^). In the gender subgroup, NHHR has a more pronounced effect on male BMD, mainly reflected in the reduction of lumbar spine and total body BMD. In the age subgroup, the negative correlation between NHHR and BMD is strongest in the younger group (18–30 years), gradually weakening in the middle-aged (31–44 years) and older groups (45–59 years). Further analysis suggests that dyslipidemia may influence bone metabolism through pathways such as inflammation and oxidative stress.

**Conclusion:**

The effect of NHHR on bone mineral density (BMD) varies by BMI, gender, and age. This study suggests that controlling NHHR levels may be a potential intervention target for bone health management, particularly for individuals with low-to-middle BMI, males, and younger populations. These findings offer a new perspective on the relationship between lipid metabolism and bone metabolism and provide scientific evidence for the development of personalized osteoporosis prevention and treatment strategies.

## 1 Introduction

Dyslipidemia refers to qualitative and quantitative changes in lipids and their metabolites in blood or other tissues, including total cholesterol, triglycerides, low-density lipoprotein (LDL), and high-density lipoprotein (HDL) (1). Osteoporosis and cardiovascular diseases (CVD) are two major global public health concerns (2, 3). Dyslipidemia can lead to various diseases such as non-alcoholic fatty liver disease (NAFLD), atherosclerosis, and CVD (4). Clinically, it has been shown that dyslipidemia in diseases like NAFLD and atherosclerosis leads to changes in bone density and bone mass, ultimately causing osteoporosis and severely affecting quality of life (5).

The relationship between lipid metabolism indicators and osteoporosis remains controversial in human studies. Most studies indicate a negative correlation between lipid biomarkers and bone mineral density (BMD) (6–10). Additionally, Ersoy et al. (11) and Lahon et al. (12) found a positive correlation between LDL-C and BMD, while Ghadiri-Anari et al. (13) suggested no correlation between lipids and BMD. Given these contradictory findings, the emerging lipid indicator Non-High-Density Lipoprotein Cholesterol to High-Density Lipoprotein Cholesterol Ratio (NHHR), as a novel lipid parameter, has not yet been studied in relation to BMD. Therefore, our investigation focused on the association between NHHR and spine bone density.

Osteoporosis is a highly prevalent condition affecting over 14 million individuals in the United States and more than 200 million people worldwide (14, 15). It is characterized by altered bone homeostasis, leading to reduced bone mass, impaired bone quality, and an increased propensity for fractures (14, 16). Hormones, cytokines, and growth factors directly or indirectly regulate bone homeostasis. Additionally, factors such as race, gender, behavior, and diet influence bone mass and the propensity to develop osteoporosis. Peak bone mass is thought to be achieved when these factors effectively interact. An imbalance in these molecular and cellular processes is believed to alter bone homeostasis, contributing to the pathophysiology of osteoporosis (17, 18). Other factors such as race, gender, behavior, and diet also impact bone homeostasis (19).

With advances in medicine and increased life expectancy, lipid-bone metabolism has become a major public health issue. The current latest study only stays on the relationship between NHHR and lumbar BMD. Therefore, it is hypothesized that there may be a correlation between NHHR and Bone mineral density at multiple sites. Understanding the relationship between lipid and bone metabolism is crucial for the intervention and management of bone density. A cross-sectional study was conducted using NHANES 2011–2018 data to explore the potential association between NHHR and BMD.

## 2 Materials and methods

### 2.1 Study population

**Figure 1:**
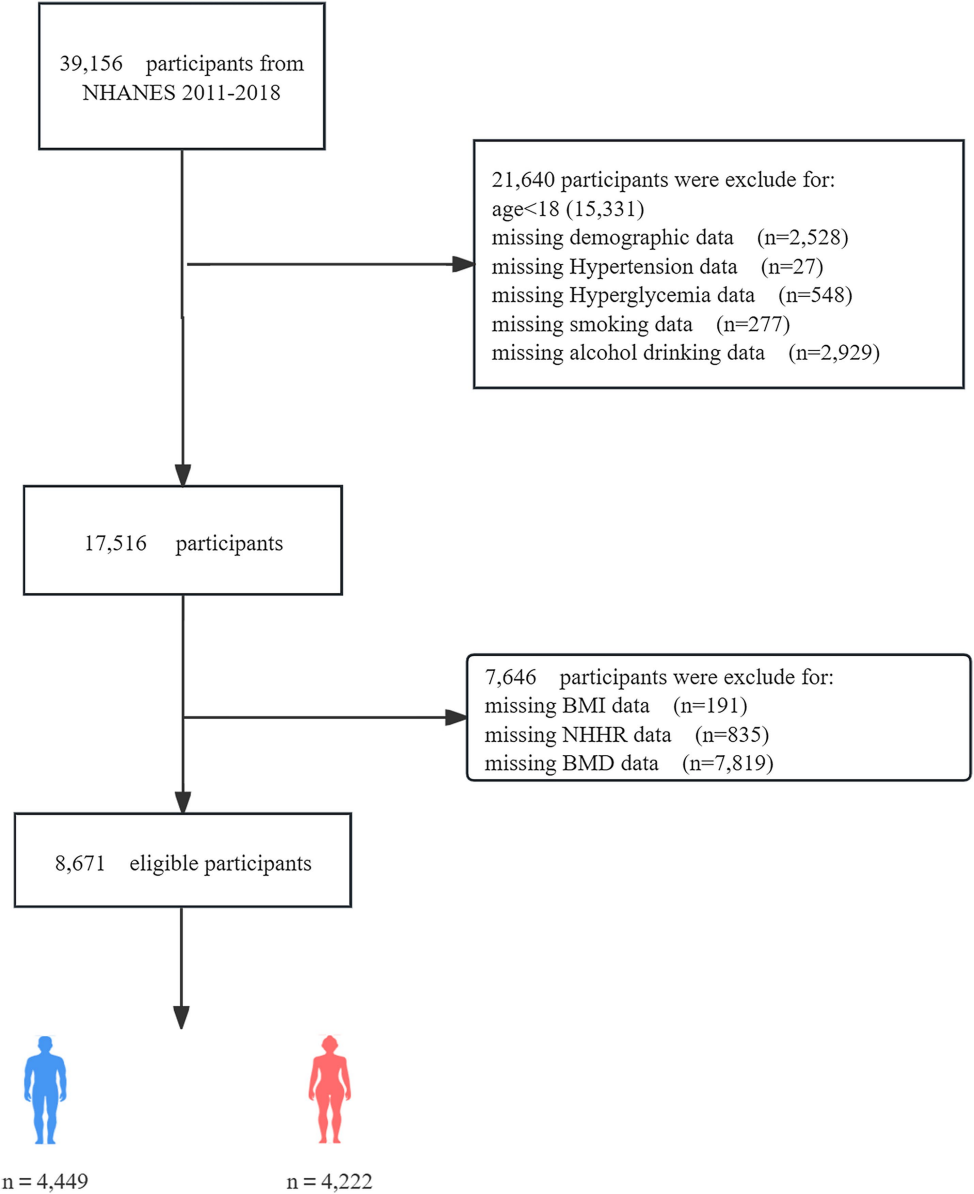
Flowchart of participant selection.

The National Health and Nutrition Examination Survey (NHANES) is a major program of the National Center for Health Statistics (NCHS) designed to assess the health and nutritional status of adults and children in the United States. The survey results are used to determine the prevalence of major diseases and risk factors for diseases, assess nutritional status, and understand its relationship with health promotion and disease prevention. The institutional review board of the NCHS authorized the survey techniques, and all NHANES participants consented to the use of their data for research. From 2011 to 2018, a total of 39,156 individuals participated in NHANES. However, after applying exclusion criteria, the sample size for this study was reduced to 8,671 eligible participants. Exclusion criteria included incomplete demographic data, missing covariate data (such as smoking status, BMI, alcohol consumption, dietary levels, hypertension history, and hyperglycemia), and lack of BMD or NHHR-related data (total cholesterol and high-density lipoprotein data). Figure 1 shows the flowchart of the screening process. The datasets generated and analyzed during the current study are available in the NHANES data^1^, or required from the corresponding author.

### 2.2 Calculation of NHHR

The total cholesterol and high-density lipoprotein data used for NHHR calculation were obtained from the laboratory data in NHANES. NHHR was calculated by subtracting high-density lipoprotein from total cholesterol and then dividing by high-density lipoprotein.

### 2.3 Study variables

To explore the relationship between NHHR and BMD, several covariates were selected for adjustment, including demographic data, lifestyle, and health status. Demographic data included age, gender, race, and income. Lifestyle factors included alcohol consumption and smoking status. Health status included measurements of BMI, hypertension, and hyperglycemia, all of which were directly obtained from questionnaires and measurement reports. Dual-energy X-ray absorptiometry was performed by qualified radiologists using Hologic QDR 4500A equipment and Apex software version 3.2 to assess BMD.

### 2.4 Statistical analysis

All statistical analyses were performed using R (version 4.2), SPSS (version 26.0), and EmpowerStats (version 2.0). Generalized linear models (GLMs) were employed to study the relationship between NHHR and BMD, given that BMD is a continuous outcome variable. And were further analyzed using a stratified regression analysis. The non-linear relationship between NHHR and BMD was explored and identified using smooth curve fitting. Threshold analysis was performed to identify the inflection point and conduct a two-piecewise linear regression model analysis on both sides of the inflection point. A *p*-value of less than 0.05 was considered statistically significant.

## 3 Results

### 3.1 Characteristics of the study population

The characteristics of the participants are shown in the Table 1. This study included 8,671 individuals, and NHHR levels were divided into tertiles. NHHR levels are significantly correlated with various health and lifestyle factors. Higher NHHR levels are associated with older age, higher BMI, and increased incidence of hypertension and hyperglycemia. Gender distribution shows a higher proportion of males in the high NHHR group. In terms of ethnicity, Non-Hispanic Whites are more prevalent in the high NHHR group. Although there are no significant differences in alcohol consumption between groups, the smoking rate is significantly higher in the high NHHR group. Overall, these results indicate that NHHR levels influence health status and lifestyle choices.

**Table 1:** Characteristics of participants.

Variables	Low (0.36–2.13)	Middle (2.13–3.24)	High (3.24–27.00)	*p*-value
NHHR	1.593 ± 0.354	2.653 ± 0.312	4.514 ± 1.491	<0.001
Age (years)	34.814 ± 12.459	37.812 ± 12.149	40.285 ± 11.096	<0.001
Gender				<0.001
Male	37.8	49.8	66.4	
Female	62.2	50.2	33.6	
Ratio of family income to poverty	2.565 ± 1.687	2.542 ± 1.657	2.400 ± 1.623	<0.001
Race				<0.001
Mexican American	11.9	14.8	19.4	
Other hispanic	8.8	10.1	11.3	
Non-hispanic white	35.7	37.0	37.2	
Non-hispanic black	25.6	19.8	14.1	
Other race—including multi-racial	17.9	18.2	18.1	
Hypertension				<0.001
Yes	15.6	22.0	26.3	
No	84.4	78.0	73.7	
Hyperglycemia				<0.001
Yes	4.4	7.0	9.8	
No	95.6	93.0	90.2	
Alcohol use				0.032
Yes	71.5	68.7	71.4	
No	28.5	31.3	28.6	
Smoking status				<0.001
Yes	33.2	37.2	45.3	
No	66.8	62.8	54.7	
BMI	25.807 ± 6.205	29.035 ± 6.847	31.011 ± 6.402	<0.001
Left arm—BMD	0.755 ± 0.093	0.770 ± 0.096	0.794 ± 0.097	<0.001
Right arm—BMD	0.777 ± 0.097	0.791 ± 0.101	0.814 ± 0.100	<0.001
Lumber spine—BMD	1.062 ± 0.153	1.032 ± 0.147	1.015 ± 0.151	<0.001
Pelvis—BMD	1.225 ± 0.164	1.244 ± 0.162	1.272 ± 0.165	<0.001
Total—BMD	1.111 ± 0.110	1.111 ± 0.109	1.117 ± 0.109	0.059

Mean +/− SD for: age, BMI, Ratio of family income to poverty, Left Arm—BMD, Right Arm—BMD, Lumber Spine—BMD, Pelvis—BMD, Total—BMD. *p*-value was calculated by weighted linear regression model. % for: Gender, Race, Hypertension, Hyperglycemia, Alcohol use, Smoking status. p value was calculated by weighted chi-square test.

### 3.2 Association between NHHR and BMD

The exposure variable NHHR was divided into low, middle, and high tertiles. The beta coefficient for the low tertile group was set to 0 and used as a reference value to evaluate the relative impact of middle and high tertile NHHR on lumbar BMD. The exposure variable NHHR was divided into three quantiles: low, medium, and high (Table 2), and three regression models (Non-adjusted, Adjust I, and Adjust II) were constructed to explore the impact of NHHR and its subgroups on BMD. The *β* coefficient of the low quantile group was set to 0 as the reference value, and the relative impact of medium and high quantiles of NHHR on BMD was assessed. The correlation between NHHR and BMD varies by anatomical site in the medium and high quantile groups. Detailed analysis in the multivariate regression model reveals a clear site-specific effect of NHHR on BMD. High NHHR is significantly positively correlated with pelvic BMD, maintaining a significant positive correlation after full adjustment. In contrast, it is consistently negatively correlated with lumbar spine BMD. For upper limb BMD, the initial positive correlation becomes non-significant or slightly negative after full adjustment. For total body BMD, high NHHR exhibits an overall negative effect. For total body BMD, high NHHR exhibits an overall negative effect, further supporting the possibility that it negatively impacts bone health through mechanisms like bone resorption or metabolic dysfunction.

**Table 2:** Association between NHHR and BMD in the 2011–2018 NHANES data.

	Non-adjusted	Adjust I	Adjust II
Left arm—BMD			
NHHR	[0.011, (0.009, 0.012), *p* < 0.00001]	[0.003, (0.002, 0.004), *p* < 0.00001]	[−0.001, (−0.002, 0.000), *p* = 0.08863]
Low	0	0	0
Middle	[0.016, (0.011, 0.021), *p* < 0.00001]	[0.003, (−0.000, 0.007), *p* = 0.06747]	[−0.005, (−0.008, −0.001), *p* = 0.00956]
High	[0.040, (0.035, 0.045), *p* < 0.00001]	[0.009, (0.005, 0.013), p < 0.00001]	[−0.005, (−0.008, −0.001), *p* = 0.01638]
Right arm—BMD			
NHHR	[0.010, (0.008, 0.011), *p* < 0.00001]	[0.001, (0.000, 0.002), *p* = 0.01665]	[−0.001, (−0.002, 0.000), *p* = 0.17738]
Low	0	0	0
Middle	[0.014, (0.009, 0.019), *p* < 0.00001]	[0.001, (−0.003, 0.004), *p* = 0.72710]	[−0.004, (−0.007, −0.000), *p* = 0.04183]
High	[0.037, (0.032, 0.042), *p* < 0.00001]	[0.004, (0.001, 0.008), *p* = 0.02415]	[−0.004, (−0.008, 0.000), *p* = 0.07315]
Lumber spine—BMD			
NHHR	[−0.012, (−0.014, −0.010), *p* < 0.00001]	[−0.006, (−0.008, −0.004), *p* < 0.00001]	[−0.008, (−0.010, −0.006), *p* < 0.00001]
Low	0	0	0
Middle	[−0.030, (−0.038, −0.023), *p* < 0.00001]	[−0.020, (−0.028, −0.013), *p* < 0.00001]	[−0.025, (−0.033, −0.017), *p* < 0.00001]
High	[−0.047, (−0.055, −0.039), *p* < 0.00001]	[−0.026, (−0.034, −0.018), *p* < 0.00001]	[−0.034, (−0.043, −0.026), *p* < 0.00001]
Pelvis—BMD			
NHHR	[0.011, (0.009, 0.014), *p* < 0.00001]	[0.014, (0.011, 0.016), *p* < 0.00001]	[0.009, (0.006, 0.011), *p* < 0.00001]
Low	0	0	0
Middle	[0.020, (0.011, 0.028), *p* < 0.00001]	[0.026, (0.018, 0.034), *p* < 0.00001]	[0.014, (0.006, 0.022), *p* = 0.00078]
High	[0.047, (0.039, 0.056), *p* < 0.00001]	[0.057, (0.049, 0.066), *p* < 0.00001]	[0.038, (0.029, 0.047), *p* < 0.00001]
Total—BMD			
NHHR	[0.001, (−0.000, 0.003), *p* = 0.08618]	[0.000, (−0.001, 0.002), *p* = 0.65782]	[−0.003, (−0.005, −0.002), *p* < 0.00001]
Low	0	0	0
Middle	[0.000, (−0.006, 0.006), *p* = 0.96387]	[−0.000, (−0.006, 0.005), *p* = 0.90435]	[−0.009, (−0.014, −0.004), *p* = 0.00049]
High	[0.006, (0.000, 0.012), *p* = 0.03707]	[0.002, (−0.003, 0.008), *p* = 0.40256]	[−0.013, (−0.019, −0.007), *p* < 0.00001]

Non-adjusted model: None. Adjust I: Gender; Age; Race. Adjust II model: Gender; Age; Race; BMI; Ratio of family income to poverty; Hypertension; Hyperglycemia; Alcohol use; Smoking status: The *β* values and 95% confidence intervals (CIs) are presented with three decimal places. If the value is displayed as 0.000, it indicates that the true value is close to zero and may have been rounded due to rounding rules. It should not be interpreted as exactly zero.

**Figure 2:**
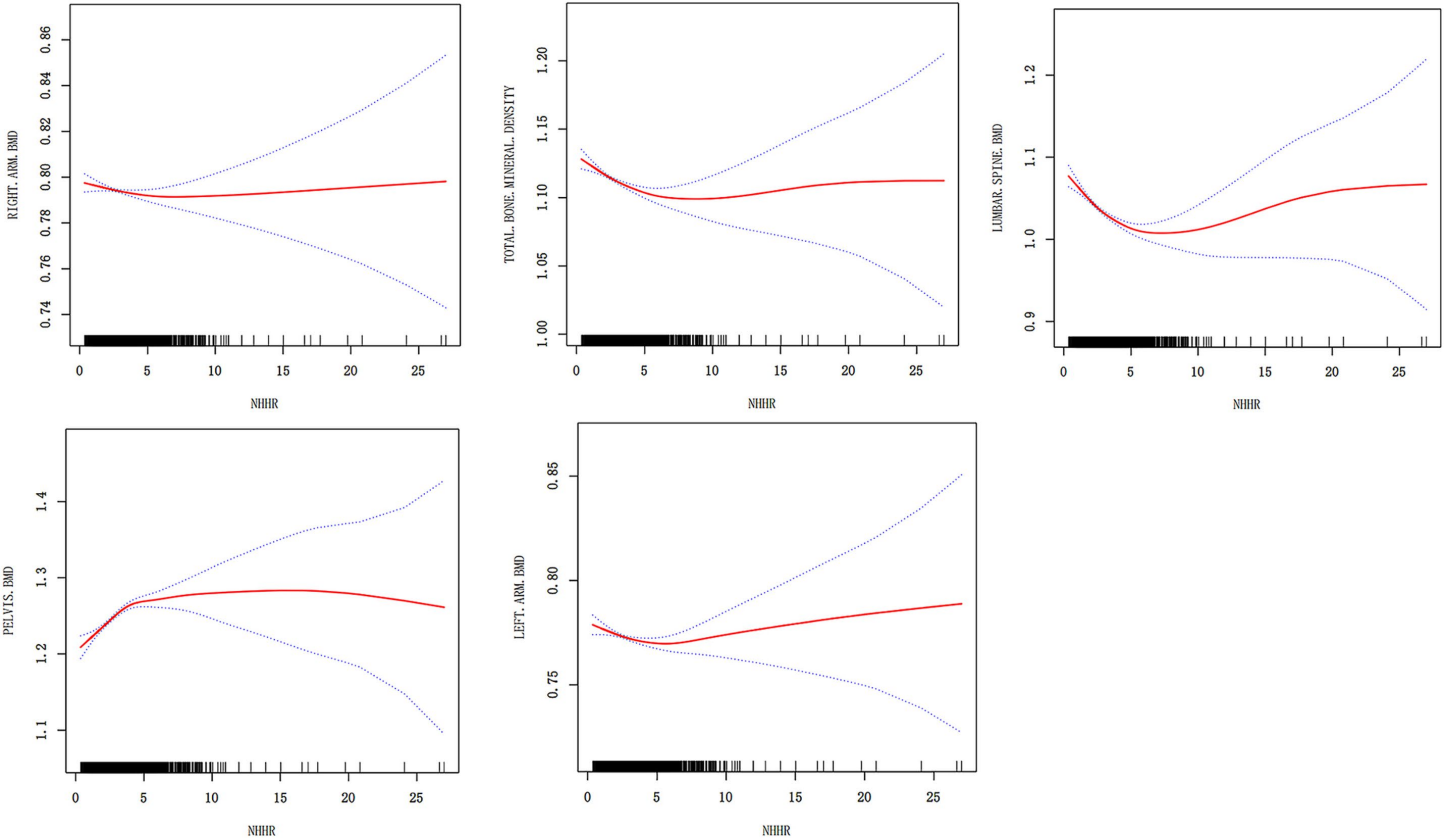
Smoothing curve fitting analysis of the relationship between NHHR and BMD at different sites. The figure shows the relationship between NHHR and different bone mineral density sites. The red curve represents the smoothed curve fit, the blue dashed line represents the 95% confidence interval, and the black short lines indicate the data distribution.

To analyze the relationship between NHHR and BMD at different sites, we conducted a smoothing curve fitting analysis (Figure 2). The results revealed a non-linear association between NHHR and BMD at various sites. Specifically, the left arm, right arm, lumbar spine, pelvis, and total BMD all exhibited significant smoothing curve trends (*p* < 0.05), indicating that changes in NHHR may have a notable impact on BMD within certain ranges. In the left arm and right arm BMD models, the smoothing curve effect was significant, with high goodness of fit (adjusted *R*^2^ values of 0.5395 and 0.5362, respectively), suggesting that NHHR has a more stable and predictable effect on limb BMD. In contrast, although the non-linear effect was significant in lumbar spine and pelvic BMD, the adjusted R^2^ values were relatively low (0.098 for lumbar spine and 0.1351 for pelvis), indicating that NHHR may be influenced by additional confounding factors at these sites. Furthermore, the smoothing curve analysis for total BMD further supports the non-linear relationship between NHHR and total body BMD (*p* < 0.05), with an adjusted *R*^2^ of 0.2118, suggesting that NHHR, as a potential metabolic marker, holds some predictive value for BMD. Regarding specific linear factors, variables such as gender, age, BMI, and race significantly affected BMD at different sites, but the extent of their influence varied by site. Some factors, such as income level, smoking, and alcohol consumption, also to further identify key inflection points or thresholds, a threshold effect analysis was performed on the smoothing curve fitting results (Table 3). In the threshold effect analysis, the linear regression results from Model I showed significant differences in the relationship between NHHR and BMD at different sites. For lumbar spine BMD and total BMD, higher NHHR levels were significantly negatively correlated with BMD, and this negative correlation remained significant after fully adjusting for confounding factors. However, for pelvic BMD, higher NHHR levels were significantly positively correlated with BMD across all models, indicating a different pattern of association compared to other BMD sites. However, the correlation between left arm and right arm BMD and NHHR was weaker and non-significant after adjustment, suggesting that upper limb BMD responds less sensitively to changes in NHHR. It is noteworthy that although the effects of NHHR on BMD differ significantly across sites, the effect in the high NHHR range is most pronounced in the lumbar spine, pelvis, and total BMD, while the effect on upper limb BMD shows lower sensitivity.

**Table 3:** Threshold effect analysis of NHHR and BMD.

	Left arm—BMD	Right arm—BMD	Lumber spine—BMD	Pelvis—BMD	Total—BMD
Model I					
Linear effect	[−0.001, (−0.002, 0.000), *p* < 0.0886]	[−0.001, (−0.002, 0.000), *p* < 0.1774]	[−0.008, (−0.010, −0.006), *p* < 0.0001]	[0.009, (0.006, 0.011), *p* < 0.0001]	[−0.003, (−0.005, −0.002), *p* < 0.0001]
Model II					
Turning point (K)	5.3	5.184	4.698	3.68	5.458
<K segment effect (1)	[−0.002, (−0.003, −0.001), *p* = 0.0020]	[−0.002, (−0.003, −0.000), *p* = 0.0243]	[−0.014, (−0.018, −0.011), *p* < 0.0001]	[0.018, (0.014, 0.023), *p* < 0.0001]	[−0.005, (−0.007, −0.004), *p* < 0.0001]
>K Segment effect (2)	[0.002, (−0.000, 0.004), *p* = 0.0969]	[0.001, (−0.001, 0.003), *P* = 0.3080]	[0.002, (−0.002, 0.007), *P* = –0.2873]	[0.001, (−0.003, 0.005), *p* = 0.6077]	[0.002, (−0.002, 0.005), *p* = 0.3774]
Difference between segment effects (2 and 1)	[0.004, (0.001, 0.007), *p* = 0.0060]	[0.003, (−0.000, 0.006), *p* = 0.0615]	[0.017, (0.011, 0.023), *p* < 0.0001]	[−0.017 (−0.024, −0.011), *p* < 0.0001]	[0.007, (0.003, 0.011), *p* = 0.0013]
Predicted value at the turning point	0.807 (0.802, 0.812)	0.825 (0.820, 0.830)	1.000 (0.994, 1.007)	1.270 (1.264, 1.277)	1.117 (1.111, 1.122)
Log-likelihood ratio test	0.006	0.061	<0.001	<0.001	0.001

Non-adjusted model: None. Adjust I: Gender; Age; Race. Adjust II model: Gender; Age; Race; BMI; Ratio of family income to poverty; Hypertension; Hyperglycemia; Alcohol use; Smoking status; The β values and 95% confidence intervals (CIs) are presented with three decimal places. If the value is displayed as 0.000, it indicates that the true value is close to zero and may have been rounded due to rounding rules. It should not be interpreted as exactly zero.

Further exploration using Model II, which incorporated breakpoint effect analysis, revealed a non-linear effect of NHHR on BMD by determining the breakpoint values (K) for each BMD site. Below the breakpoint value, increases in NHHR had a significant impact on BMD at all sites, with significant changes observed in lumbar spine, pelvic, and total BMD within the low NHHR range. Specifically, the negative correlation for lumbar spine BMD was most pronounced when NHHR < K, while pelvic and total BMD showed a positive correlation. However, once NHHR exceeded the breakpoint, the effect on BMD diminished or became non-significant, suggesting that after reaching a certain level, changes in NHHR lead to stable or negligible changes in BMD. Notably, the breakpoint effects were more prominent in the lumbar spine and pelvic regions, indicating that weight-bearing sites are more sensitive to metabolic abnormalities, potentially due to their metabolic activity and load-bearing functions. The likelihood ratio test results further support the validity of Model II, with all breakpoint effects being statistically significant (*p* < 0.05) except for right arm BMD (*p* = 0.061), indicating that NHHR changes exhibit clear threshold effects at different BMD sites, with the effect being more significant in the low NHHR range. This analysis provides new insights into the impact of NHHR on BMD, suggesting that sensitivity to metabolic abnormalities may vary by bone site. Controlling NHHR below specific threshold levels may help improve bone health and provide evidence for prevention and treatment strategies. Showed significant differences.

### 3.3 Stratified analysis of the relationship between NHHR and BMD: insights into modulatory effects and population-specific variations

**Figure 3:**
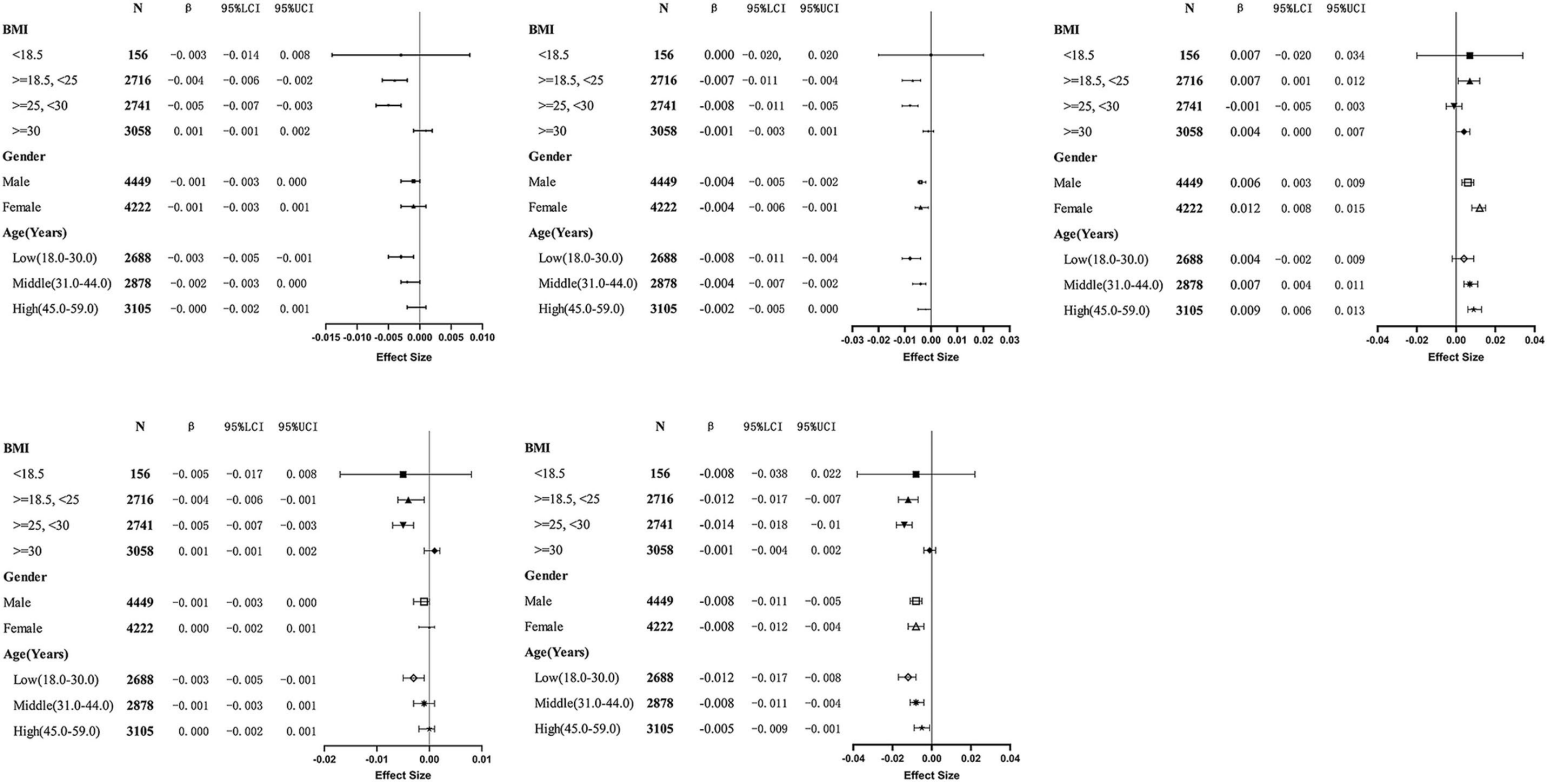
Forest plot of stratified analysis of the relationship between NHHR and bone mineral density (BMD) across different BMI, gender, and age groups. This figure illustrates the effects of different gender (Male and Female), age groups [Low (18–30.0), Middle (31–44.0), High (45–59.0)], and BMI categories (<18.5, 18.5 - < 25, 25 - < 30, ≥30) on bone mineral density (BMD) at various body sites (Left Arm, Right Arm, Lumbar Spine, Pelvis, Total). The chart includes statistical data such as 95% confidence intervals (95% LCI and 95% UCI), *β* values (B), sample size (N), and effect size.

The choice of age, gender, and BMI as stratification factors is based on their potential to modulate the relationship between NHHR and BMD. For example, with increasing age, bone density naturally declines, particularly in individuals at higher risk for osteoporosis. Additionally, population characteristics have shown that women generally have lower bone density than men and are more susceptible to changes in hormone levels, especially post-menopause. Furthermore, BMI, an important metabolic indicator that reflects an individual’s body fat percentage, is widely acknowledged for its relationship with bone density. Age, gender, and BMI are crucial potential confounders or effect modifiers when examining the relationship between NHHR and BMD. Stratified analysis considering these variables offers a more precise insight into the true association between NHHR and BMD, and explores the potential differential effects of NHHR on BMD in different populations. Stratified analysis by gender, age, and BMI was conducted to examine the relationship between NHHR and BMD at different anatomical sites (Table 4), and draw the related forest map (Figure 3). The results indicated the following: BMI Stratification: In the BMI < 18.5 group (underweight), no significant relationship between NHHR and BMD was observed (all *p*-values >0.05). In the BMI 18.5–25 group (normal weight), NHHR showed a significant negative correlation with left arm BMD (*β* = −0.004, *p* = 0.0004), right arm BMD (*β* = −0.004, *p* = 0.0021), and lumbar BMD (*β* = −0.012, *p* < 0.0001). This suggests that in individuals with normal weight, an increase in NHHR is associated with reduced bone density, especially in the upper limbs and lumbar spine. In the BMI ≥ 25 and < 30 group (overweight), NHHR also exhibited a significant negative correlation, particularly in the left arm (*β* = −0.005, *p* < 0.0001), right arm (*β* = −0.005, *p* < 0.0001), and lumbar BMD (*β* = −0.014, *p* < 0.0001). This suggests that metabolic abnormalities associated with overweight may exacerbate bone density loss. In the BMI ≥ 30 group (obese), while NHHR showed a weak negative correlation with BMD, the effect was minimal and not statistically significant (higher *p*-values), potentially due to the metabolic characteristics (e.g., insulin resistance, chronic inflammation) and bone physiology (e.g., increased skeletal load) typical of obesity. Gender Stratification: In the male population, NHHR showed a significant negative correlation with lumbar BMD (*β* = −0.008, *p* < 0.0001) and total BMD (*β* = −0.004, *p* = 0.0004), while the relationship with left arm (*β* = −0.001, *p* = 0.0613) and right arm BMD (*β* = −0.001, *p* = 0.0761) was not significant. This indicates that NHHR increase is associated with reduced lumbar and total BMD in males, but has a minimal effect on the upper limbs. In females, NHHR showed significant negative correlations with lumbar BMD (*β* = −0.008, *p* < 0.0001) and total BMD (*β* = −0.004, *p* = 0.0037), while the relationship with left and right arm BMD was not significant (higher *p*-values). Age Stratification: In the younger group (18–29 years), NHHR showed a significant negative correlation with lumbar BMD (*β* = −0.012, *p* < 0.0001) and total BMD (*β* = −0.008, p < 0.0001), with weaker associations in the upper limbs. This suggests that younger individuals’ bone density is more sensitive to metabolic factors, especially with more pronounced changes in lumbar and total BMD. In the middle-aged group (30–43 years), NHHR continued to show a significant negative correlation with lumbar BMD (*β* = −0.008, p < 0.0001) and total BMD (*β* = −0.004, *p* = 0.0006), with weaker associations in the upper limbs. In the older group (44–59 years), while there was a negative correlation with lumbar BMD (*β* = −0.005, *p* = 0.0087), the changes in total BMD (*β* = −0.002, *p* = 0.1155) and upper limbs BMD were not significant, possibly due to the multifactorial nature of bone density changes in older individuals, such as reduced bone remodeling and metabolic slowing. Notably, NHHR exhibited a positive correlation with pelvic BMD in the normal BMI group (*β* = 0.007, *p* = 0.0146) and obese group (*β* = 0.004, *p* = 0.0341), with a stronger association in females (*β* = 0.012, *p* < 0.0001) compared to males (*β* = 0.006, *p* = 0.0002). Age stratification revealed a significant association in the middle-aged (*β* = 0.007, *p* = 0.0002) and older (*β* = 0.009, *p* < 0.0001) groups, but not in the younger group.

**Table 4:** Stratified analysis of the relationship between NHHR and bone mineral density (BMD) across different BMI, gender, and age groups.

	*N*	Left arm—BMD	Right arm—BMD	Lumber spine—BMD	Pelvis—BMD	Total—BMD
BMI						
<18.5	156	[−0.003, (−0.014, 0.008), *p* = 0.5706]	[−0.005, (−0.017, 0.008), *p* = 0.4610]	[−0.008, (−0.038, 0.022), *p* = 0.6017]	[0.007, (−0.020, 0.034), *p* = 0.5956]	[−0.000, (−0.020, 0.020), *p* = 0.9831]
> = 18.5, <25	2,716	[−0.004, (−0.006, −0.002), *p* = 0.0004]	[−0.004, (−0.006, −0.001), *p* = 0.0021]	[−0.012, (−0.017, −0.007), *p* < 0.0001]	[0.007, (0.001, 0.012), *p* = 0.0146]	[−0.007, (−0.011, −0.004), *p* < 0.0001]
> = 25, <30	2,741	[−0.005, (−0.007, −0.003), *p* < 0.0001]	[−0.005, (−0.007, −0.003), *p* < 0.0001]	[−0.014, (−0.018, −0.010), *p* < 0.0001]	[−0.001, (−0.005, 0.003), *p* = 0.6986]	[−0.008, (−0.011, −0.005), *p* < 0.0001]
> = 30	3,058	[0.001, (−0.001, 0.002), *p* = 0.3026]	[0.001, (−0.001, 0.002), *p* = 0.4744]	[−0.001, (−0.004, 0.002), *p* = 0.5832]	[0.004, (0.000, 0.007), *p* = 0.0341]	[−0.001, (−0.003, 0.001), *p* = 0.3305]
Gender						
Male	4,449	[−0.001, (−0.003, 0.000), *p* = 0.0613]	[−0.001, (−0.003, 0.000), *p* = 0.0761]	[−0.008 (−0.011, −0.005), *p* < 0.0001]	[0.006, (0.003, 0.009), *p* = 0.0002]	[−0.004, (−0.005, −0.002), *p* = 0.0004]
Female	4,222	[−0.001, (−0.003, 0.001), *p* = 0.2202]	[−0.000, (−0.002, 0.001), *p* = 0.5788]	[−0.008, (−0.012, −0.004), *p* < 0.0001]	[0.012, (0.008, 0.015), *p* < 0.0001]	[−0.004, (−0.006, −0.001), *p* = 0.0037]
Age (years)						
Low (18–29 years)	2,688	[−0.003, (−0.005, −0.001), *p* = 0.0132]	[−0.003, (−0.005, −0.001), *p* = 0.0156]	[−0.012, (−0.017, −0.008), *p* < 0.0001]	[0.004 (−0.002, 0.009), *p* = 0.1887]	[−0.008, (−0.011, −0.004), *p* < 0.0001]
Middle (30–43 years)	2,878	[−0.002, (−0.003, −0.000), *p* = 0.0370]	[−0.001, (−0.003, 0.001), *p* = 0.2167]	[−0.008, (−0.011, −0.004), *p* < 0.0001]	[0.007, (0.004, 0.011), *p* = 0.0002]	[−0.004, (−0.007, −0.002), *p* = 0.0006]
High (44–59 years)	3,105	[−0.000, (−0.002, 0.001), *p* = 0.6816]	[−0.000, (−0.002, 0.001), *p* = 0.6251]	[−0.005, (−0.009, −0.001), *p* = 0.0087]	[0.009, (0.006, 0.013), *p* < 0.0001]	[−0.002, (−0.005, 0.000), *p* = 0.1155]

The *β* values and 95% confidence intervals (CIs) are presented with three decimal places. If the value is displayed as 0.000, it indicates that the true value is close to zero and may have been rounded due to rounding rules. It should not be interpreted as exactly zero.

In conclusion, with increasing BMI, the negative impact of NHHR on BMD becomes more pronounced, especially in individuals with normal weight and those who are overweight, while the effect is weaker in the obese population. Males are more sensitive to NHHR changes, particularly in lumbar and total BMD, while females are influenced by other physiological factors. Younger individuals exhibit greater sensitivity to NHHR changes in bone density, with a more gradual response in the middle-aged group and a more complex interaction of factors in the elderly.

## 4 Discussion

This study, based on the NHANES database, systematically investigates the association between NHHR and BMD, with detailed stratified analysis according to different BMI, gender, and age groups. The results demonstrate stratified associations between NHHR and BMD at various anatomical sites. Specifically, in individuals with BMI < 25 kg/m^2^ (underweight to normal weight), NHHR was significantly negatively correlated with BMD. Gender stratification revealed that men showed a more pronounced negative correlation across multiple BMD sites. Age stratification indicated that NHHR had a particularly strong effect on bone density in younger individuals, with this correlation weakening as age increased. In fully adjusted models, for each unit increase in NHHR, L-BMD decreased by 0.008 units, total BMD decreased by 0.003 units, and pelvic BMD increased by 0.009 units (*p* < 0.01). The left and right arm BMD decreased by 0.001 units, which was not statistically significant (*p* > 0.05).

Osteoporosis has a close relationship with lipid markers and lipid metabolism. Triglycerides, total cholesterol, high-density lipoprotein (HDL), and low-density lipoprotein (LDL) are important lipid metabolic indicators, reflecting the lipid metabolic status to some extent. In recent years, the interplay between glucose, lipids, and bone metabolism has garnered attention in scientific research. Several studies have found that high cholesterol diets and elevated triglyceride levels are associated with an increased risk of osteoporosis. Osteoporosis treatments such as bisphosphonates and calcium supplements can cause abnormal triglyceride metabolism in adipose tissue. Moreover, moderate reduction of triglyceride levels in postmenopausal mouse models can improve BMD (20–24). Hyperglycemia inhibits bone formation and promotes adipogenesis, while dysregulated lipid metabolism is a key driver of osteoporosis in diabetic patients (25–27). In diabetic individuals, total cholesterol, triglycerides, and LDL levels are negatively correlated with BMD (28). Furthermore, obesity increases the risk of coronary artery disease, hypertension, type 2 diabetes, and osteoporosis, typically characterized by elevated triglycerides and low HDL cholesterol (29–31).

At the molecular level, the role of lipids in osteoporosis is complex. Lipids can influence the differentiation of mesenchymal stem cells (MSCs) through various signaling pathways, contributing to the onset and progression of osteoporosis. Oxidized lipids bind to and activate the PPARγ signaling pathway, which interacts with CCAAT/enhancer-binding proteins, inhibiting osteoblast differentiation and promoting fat accumulation while suppressing bone formation. Additionally, some oxidized lipids act on bone marrow stromal cells, inducing the expression of epidermal growth factor, which further enhances alkaline phosphatase activity and osteoblast differentiation (5, 32). HDL and LDL, as central components of lipid metabolism, have been closely linked to changes in BMD. High plasma cholesterol levels can downregulate the Wnt signaling pathway, affecting MSC differentiation into osteoblasts. It can also alter Runx2 levels through changes in the bone morphogenetic protein/transforming growth factor *β*/Wnt pathway, thus influencing osteoblast proliferation and maturation and disrupting the balance of bone remodeling between osteoblasts and osteoclasts (23, 33, 34). HDL is considered to have anti-inflammatory and antioxidant effects, potentially promoting bone health by inhibiting osteoclast formation and function, as well as stimulating osteoblast differentiation and mineralization (35, 36). In contrast, LDL and its oxidized form (OxLDL) are typically considered pro-inflammatory, possibly regulating bone metabolism negatively by enhancing osteoclast activity or inhibiting osteoblast differentiation (37, 38).

Animal studies and *in vitro* research have further revealed the direct effects of HDL and LDL on bone cells. For instance, HDL inhibits the activation of the NF-κB signaling pathway, reducing the expression of osteoclast-related genes (39). Additionally, ApoA-1 in HDL has been shown to promote osteoblast proliferation and differentiation by activating the Wnt/*β*-catenin signaling pathway (40). In contrast, LDL and OxLDL promote ROS production and imbalance the RANKL/OPG axis, significantly enhancing osteoclast differentiation while inhibiting osteoblast activity (41, 42). These effects are particularly pronounced in the pathological mechanisms of osteoporosis, especially in populations with lipid metabolism disorders. Further molecular biological studies suggest that the role of HDL and LDL in bone metabolism is not limited to lipid regulation but involves multiple cellular signaling pathways. For example, HDL enhances osteogenic differentiation of MSCs by activating the AMPK (5’ AMP-activated protein kinase) pathway, while inhibiting adipogenesis and oxidative stress to protect bone health (43). HDL also increases the sensitivity of bone marrow stromal cells to insulin, promoting their differentiation into the osteoblast lineage, thus enhancing BMD (44). In contrast, LDL activates the JNK (c-Jun N-terminal kinase) and ERK (extracellular signal-regulated kinase) signaling pathways, promoting osteoclast differentiation and inhibiting osteoblast mineralization (45, 46). Notably, OxLDL activates the RhoA/ROCK signaling pathway, increasing osteoclast resorptive activity and inhibiting the expression of bone formation-related genes, further exacerbating osteoporosis (47). These studies not only deepen our understanding of the specific roles of HDL and LDL in bone metabolism but also provide potential molecular targets for future interventions in lipid metabolism.

As a novel lipid parameter, NHHR has been significantly associated with abdominal aortic aneurysm, suicidal ideation, coronary atherosclerotic heart disease, myocardial infarction, and non-alcoholic fatty liver disease. Moreover, there exists a potential clinical link between lipid profiles and bone metabolism: elevated NHHR levels can lead to decreased lumbar spine BMD, thereby contributing to a range of bone health issues (48–54). The results indicate that NHHR demonstrates varying threshold effects at different body sites, and the relationship between NHHR and BMD’s threshold effect is likely influenced by its multifaceted roles in regulating lipid metabolism, inflammatory states, and bone remodeling signaling pathways. The threshold effect of NHHR may arise from its dual impact on the balance between osteoblasts and osteoclasts due to lipid metabolism. Lower NHHR levels might reflect higher HDL levels, which protect bone health by reducing inflammation, inhibiting osteoclast differentiation and activity, and enhancing osteoblast differentiation (55, 56). However, when NHHR exceeds a certain threshold, the accumulation of LDL and its oxidized form (OxLDL) can induce reactive oxygen species (ROS) production, activate the NF-κB and RANKL/OPG pathways, significantly promoting osteoclast-mediated bone resorption, while suppressing osteoblast mineralization, thereby leading to a reduction in BMD (57, 58).

Furthermore, the variation in NHHR thresholds across different skeletal sites may be related to differences in local bone metabolic biomechanics and blood supply. For example, lumbar and pelvic BMD, which are subjected to higher mechanical loads, may exhibit higher osteoblast activity and better tolerance to lipid metabolic disturbances, suggesting a higher NHHR threshold for these sites (59). In contrast, sites like the trunk and distal limbs have lower blood supply and are more sensitive to changes in lipid metabolism, indicating lower NHHR thresholds (60, 61). Additionally, local lipid deposition and secretion of adipokines may further exacerbate the differential responses of various skeletal sites to lipid metabolic changes. For instance, leptin and adiponectin secreted by adipocytes in the bone marrow cavity play significant roles in regulating bone metabolism, and their concentrations and mechanisms may differ across sites, potentially leading to heterogeneous relationships between NHHR and BMD (62, 63).

The threshold effect between NHHR and BMD may stem from the bidirectional regulation of bone metabolism by lipid metabolism through inflammation, oxidative stress, and osteoclast signaling pathways. The differences in thresholds between various skeletal sites are likely determined by a combination of local blood supply, biomechanical characteristics, and the distribution and function of adipokines. Future studies are needed to further elucidate these physiological mechanisms to optimize strategies for the prevention and treatment of osteoporosis related to lipid metabolism.

## 5 Conclusion

This study utilized a large, representative sample, which enhanced the statistical power and generalizability of the results. BMD was measured with high precision using dual-energy X-ray absorptiometry (DXA), ensuring the accuracy and reliability of the data. Additionally, a systematic analysis across multiple BMD sites (including the left arm, right arm, lumbar spine, pelvis, and total body) further validated the systemic impact of NHHR on bone health. Compared to previous studies, this research delineated the heterogeneous effects of NHHR on BMD across different subgroups by stratifying by BMI, sex, and age, providing new insights for precise bone health management.

However, there are some limitations to this study. Its cross-sectional design precludes the establishment of a causal relationship between NHHR and BMD, showing only a correlation. The measurement of NHHR was based on a single lipid level assessment, and the use of lipid-lowering medications by participants was not recorded. Additionally, the sample was primarily drawn from one region, which may limit the generalizability to other populations; further validation across diverse regions and populations is needed. Future studies should involve repeated measurements, multi-region validation, and attempt to control potential confounders (For example, the specific amount of exercise, female physiological cycle, etc.) in the relationship between NHHR and BMD to better define the causal link between NHHR and BMD.

## Data Availability

Publicly available datasets were analyzed in this study. This data can be found here: the datasets generated and analyzed during the current study are available in the NHANES data (www.cdc.gov/nchs/nhanes), or required from the corresponding author.

## Footnotes

^1^ www.cdc.gov/nchs/nhanes
